# Proteomic Analysis of *Vibrio parahaemolyticus*-Stimulated *Pinctada martensii* Proteins for Antimicrobial Activity, Potential Mechanisms, and Key Components

**DOI:** 10.3390/antibiotics13111100

**Published:** 2024-11-19

**Authors:** Haisheng Lin, Weiqiang Shen, Bei Luo, Wenhong Cao, Xiaoming Qin, Jialong Gao, Zhongqin Chen, Huina Zheng, Bingbing Song

**Affiliations:** 1 College of Food Science and Technology, Guangdong Ocean University, Zhanjiang 524088, China; linhs@gdou.edu.cn (H.L.); shenweiqiang1@stu.gdou.edu.cn (W.S.); luobei1@stu.gdou.edu.cn (B.L.); cwenhong@gdou.edu.cn (W.C.); xiaoming0502@21cn.com (X.Q.); gaojl@gdou.edu.cn (J.G.); chenzhongqin@gdou.edu.cn (Z.C.); zhenghn@gdou.edu.cn (H.Z.); 2 National Research and Development Branch Center for Shellfish Processing (Zhanjiang), Guangdong Ocean University, Zhanjiang 524088, China; 3 Guangdong Provincial Key Laboratory of Aquatic Product Processing and Safety, Guangdong Ocean University, Zhanjiang 524088, China; 4 Guangdong Province Engineering Laboratory for Marine Biological Products, Guangdong Ocean University, Zhanjiang 524088, China

**Keywords:** *Pinctada martensii*, antimicrobial proteins, proteomics, mechanism, components

## Abstract

**Background:** Bacterial infections are a major challenge in food processing and public health, and there is an urgent need to develop novel antimicrobial agents. **Objectives:** The purpose of this study is to investigate the potential mechanism and key components of *Pinctada martensii* antimicrobial proteins (Pm-Aps) to provide a theoretical basis for the development of novel antimicrobial agents. **Methods:** The researchers used *Vibrio parahaemolyticus* (VP) to stimulate *Pinctada martensii*, extracted the antimicrobial proteins, and analyzed their antimicrobial activities, potential mechanisms of action, and key components using proteomics. **Results:** The results showed that the antimicrobial activity of Pm-Aps, with broad-spectrum antimicrobial effects, was significantly enhanced after VP stimulation. This was associated with the upregulation of *LAAO*, *CHDH*, *TLR2*, *ATG16L1*, *BAK*, *CLCA4*, and *CASP8* and the downregulation of *MCM3*, *MCM5*, *DTYMK*, *PLK1*, *FBXO6*, *LPCAT3*, *GST*, *LAMTOR5*, *CYP17A*, *CTSA*, and *RRM1*. It is hypothesized that these proteins may inhibit bacterial growth and multiplication by activating immune-related signaling pathways, inhibiting DNA replication and repair, and inducing apoptosis and autophagy. Furthermore, it was found that *LAAO* may be a key component of the antimicrobial action of Pm-Aps, killing bacteria by catalyzing the oxidation of amino acids to produce hydrogen peroxide (H_2_O_2_). **Conclusions:** These results strongly suggest that Pm-Aps is an effective antimicrobial protein, and it is expected that new *LAAO* can be obtained from Pm-Aps.

## 1. Introduction

Bacterial infections not only cause inflammatory diseases [1,2] but also cause serious losses in aquaculture and food processing [3,4]. For example, *Vibrio parahaemolyticus*, *Bacillus cereus*, *Staphylococcus aureus*, *Escherichia coli*, and *Listeria monocytogenes* have been associated with food poisoning incidents and are particularly hazardous to the quality and safety of raw meat products, especially aquatic products [5]. *Vibrio parahaemolyticus*, *Streptococcus agalactiae*, and *Streptococcus iniae* cause diseases in aquaculture, resulting in significant economic losses to the aquaculture industry [6,7]. *Pseudomonas aeruginosa* can infect immunocompromised individuals, causing disease [8]. Currently, antibiotics are one of the most commonly used antimicrobial agents to fight bacterial infections with a broad-spectrum antimicrobial effect. However, the misuse of antibiotics has led to increasing bacterial resistance, making them less effective and posing a huge challenge [9]. Thus, there is an urgent need for a novel antimicrobial agent with high anti-drug-resistant bacterial activity.

Natural antimicrobial agents include alkaloids, glycosides, flavonoids, bacteriocins, antimicrobial peptides, and antimicrobial proteins [10,11,12]. Among them, antimicrobial proteins have different mechanisms of action than antibiotics. Most of the antimicrobial proteins inhibit the growth of bacterial cells through membrane destabilization and membrane pore formation [13,14]. In addition to targeting membranes, antimicrobial proteins also target various intracellular biosynthetic processes in bacteria, such as the biosynthesis of proteins, DNA, and RNA [15]. Furthermore, antimicrobial proteins defend against bacterial infection by inducing apoptosis and autophagy as well as regulating the secretion of relevant inflammatory factors [16]. Hence, it is difficult for bacteria to develop resistance to them. Meanwhile, natural antimicrobial proteins are characterized by safety, high stability, and high biocompatibility, which have the potential to become an alternative to antibiotics [17]. To date, the extraction of natural proteins from nature for antimicrobial use has proven to be useful. For example, lactoferrin has antimicrobial activity against a variety of bacteria, including flesh-eating bacteria, *Listeria monocytogenes*, and *Escherichia coli* [18]. Antimicrobial peptides from *Litopenaeus vannamei* inhibit the growth of gram-negative and gram-positive bacteria [19]. Currently, antimicrobial proteins play an important role in the field of antimicrobial agents due to their excellent antimicrobial functions.

Animals produce a number of humoral defense factors, such as antimicrobial proteins, when stimulated by microorganisms or toxic substances in their natural environment. Therefore, stimulation to induce the production of antimicrobial proteins in animals and to explore their antimicrobial mechanisms is commonly used [20,21]. *Pinctada martensii*, as one of the major pearl brooding shells, is often infected by *Vibrio parahaemolyticus* during the insertion of nuclei for pearl brooding, causing its gill tissues to secrete large amounts of antimicrobial proteins [22]. The trypsin serine protease of *Pinctada martensii* has been found to kill bacteria by disrupting their cell membranes [23], and the histone 2A-derived antimicrobial peptide exerts its antimicrobial activity through membrane cleavage [24]. However, the activity of the antimicrobial proteins of *Pinctada martensii* is not high, and the mechanism of action is not obvious. In addition, the gill tissue, which is often discarded as a by-product after the removal of beads from *Pinctada martensii*, can be utilized for high-value processing by using it to extract antimicrobial proteins.

Therefore, in this study, *Vibrio parahaemolyticus* was injected to imitate the microbial infections of *Pinctada martensii* in its natural environment, to induce the production of antimicrobial proteins in its gill tissues, and to determine its antimicrobial activity. We also analyzed the expression of different proteins in the gill tissues before and after induction using proteomics to explore the potential mechanisms associated with the antimicrobial effects of *Pinctada martensii* antimicrobial proteins (Pm-Aps) and to identify the effective key components. The present study provides a theoretical basis for the development of Pm-Aps in the field of natural antimicrobial agents. Meanwhile, it provides some new ideas for the study of inducing the production of antimicrobial proteins using immunostimulation methods.

## 2. Results

### 2.1. Effect of VP-Stimulated Infection on Pm-Aps Activity

**Figure 1:**
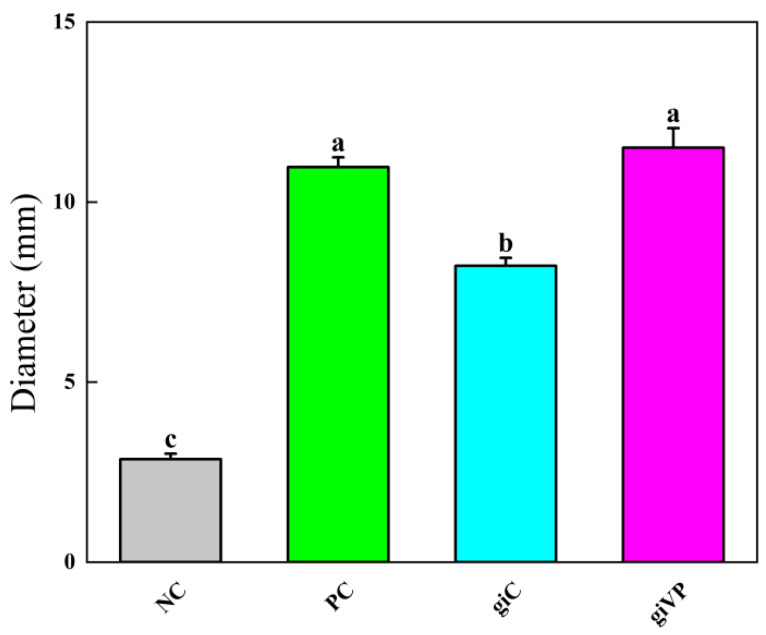
The effect of VP-stimulated infection on Pm-Aps activity. Normal control (NC): sterile PBS; positive control (PC): 1.5% H_2_O_2_; control group (giC): no VP-stimulated Pm-Aps; experimental group (giVP): VP-stimulated Pm-Aps. Different alphabets indicate significant differences (*p* < 0.05).

The inhibitory activity was determined using equal concentrations of giVP- and giC-extracted Pm-Aps solutions, and the results are shown in Figure 1. giVP produced an inhibitory circle with a diameter of (11.51 ± 0.54) mm, and the diameter of the inhibitory circle of giC was (8.23 ± 0.22) mm, which indicated that stimulation of the infection by VP resulted in a significant increase in the activity of Pm-Aps, and it was greater than that of the PC group.

### 2.2. Antimicrobial Spectrum and Antimicrobial Activity of Pm-Aps

We found that Pm-Aps had an inhibitory effect on VP, and the activity of Pm-Aps produced after induction by VP stimulation was enhanced. To further understand the antimicrobial effect of giVP-extracted Pm-Aps, its antimicrobial spectrum and antimicrobial activity were determined. As shown in Table 1, Pm-Aps produced a zone of inhibition against all bacteria tested. The strongest inhibitory effect of Pm-Aps was observed against VP, while the weakest inhibitory effect was observed against EC. The antimicrobial activity of Pm-Aps was determined using an IT, which showed that Pm-Aps had the highest antimicrobial activity against VP (IT = 16) and the lowest antimicrobial activity against BC, EC, PA, SE, and SI (IT = 4).

### 2.3. Differential Protein Expression Screening of Pm-Aps Before and After VP-Stimulated Infection

**Figure 2:**
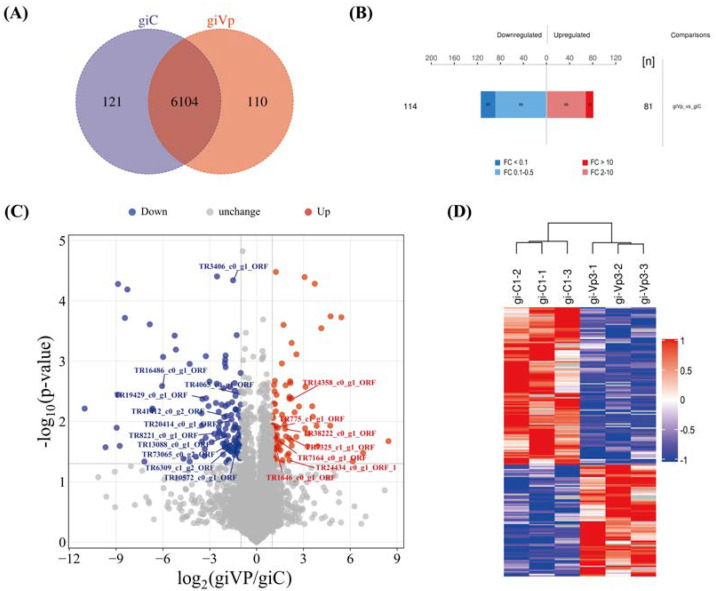
Screening of DEPs before and after VP-stimulated infection. (**A**) Venn diagram of proteins identified in giVP and giC. (**B**) Histogram of DEPs results. Upregulated DEPs are in red, and downregulated DEPs are in blue. (**C**) Volcano map of DEPs. Red dots are significant upregulated DEPs (FC > 2.0 and *p* < 0.05), blue dots are prominent downregulated DEPs (FC < 0.5 and *p* < 0.05), and gray dots are proteins with no differential change. The details of the labeled proteins are shown in Table 2 and Table 3. (**D**) Hierarchical cluster analysis heatmap of DEPs. A redder color means that the protein is upregulated for significance in this sample, and a bluer color means that the protein is downregulated for significance in this sample. Control group (giC): no VP-stimulated Pm-Aps; experimental group (giVP): VP-stimulated Pm-Aps.

The overlap in protein identification between giVP and giC is shown in Figure 2A. The total number of proteins identified between giVP and giC was 6335, of which 6104 proteins overlapped. There were also 121 proteins that were specific to giC, while a total of 110 specific proteins were identified for giVP. Differentially expressed proteins (DEPs) were screened by the expression fold change (FC) and *p* < 0.05, with an FC > 2.0 being significantly upregulated and an FC < 0.5 significantly downregulated, and the results are shown in Figure 2B,C. Compared with giC, giVP had a total of 195 DEPs, including 81 significantly upregulated proteins and 114 significantly downregulated proteins, of which 13 proteins had an FC > 10 and 25 proteins had an FC < 0.1. In addition, Table 2 and Table 3 list the major DPEs associated with antimicrobial activity. L-amino-acid oxidase-like isoform X1 (*LAAO*) expression was the most upregulated, and ribonucleoside-diphosphate reductase large subunit-like (*RRM1*) expression was the most downregulated. Cluster analysis of the DEPs (Figure 2D) showed that giVP and giC were similarly expressed within the samples and dramatically different compared to their expression between the samples, respectively.

### 2.4. GO Functional Annotation and Enrichment Analysis

**Figure 3:**
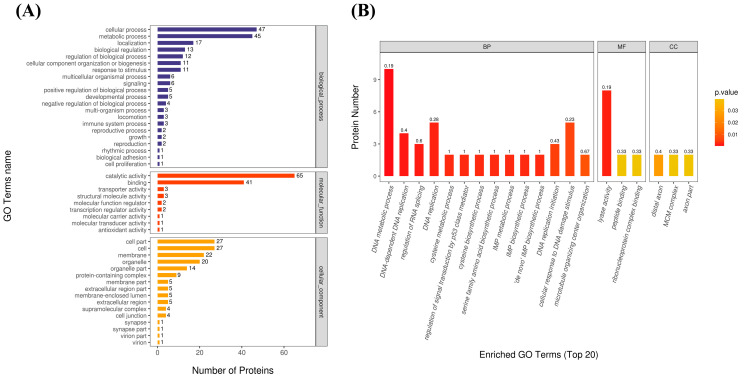
GO functional annotation and enrichment analysis of DEPs. (**A**) GO annotations of statistical analysis of DEPs. (**B**) GO functional enrichment of DEPs. A BP is a biological process, an MF is a molecular function, and a CC is a cellular component; the number Rich factor, above the bars, is the number of relevant differential proteins as a proportion of all characterized proteins for that GO term.

GO functional analysis enables a more comprehensive analysis of the localization, function, and biological pathways involved in DEPs in organisms. The results of the GO function annotation are shown in Figure 3A. DEPs were annotated in biological processes (BPs), such as cellular processes and metabolic processes. The molecular functions (MFs) annotated included catalytic activity, binding, and so on. In addition, DEPs were annotated on cellular component (CC) terms such as cell part, cell, cell membrane, and organelle.

To further analyze the biological pathways by which DEPs exert their antimicrobial functions, all DEPs were enriched for analysis, including BPs, MFs, and CCs, at *p* < 0.05, and Fisher’s exact test. In Figure 3B, DEPs were mainly enriched on BPs in DNA metabolic processes, DNA-dependent DNA replication, the regulation of RNA splicing, DNA replication, and the regulation of signal transduction by p53 class mediator. Among them, the DNA metabolic process showed the highest enrichment. The MFs included lyase activity, peptide binding, and ribonucleoprotein complex binding. Furthermore, lyase activity was enriched to a higher degree than others. In CC GO terms, DEPs were mainly enriched in distal axons, MCM complexes, and axon parts.

### 2.5. KEGG Annotation and Enrichment Analysis

**Figure 4:**
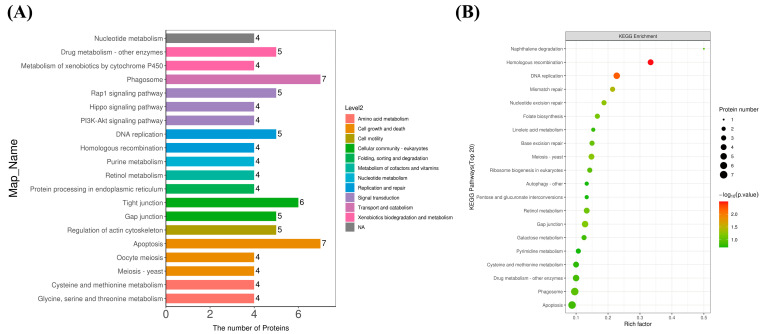
KEGG annotation and enrichment analysis of DEPs. (**A**) KEGG annotations of statistical analysis of DEPs. (**B**) KEGG pathway enrichment of DEPs. The rich factor is the ratio of the number of relevant differential proteins to the number of characterized proteins in the pathway.

The KEGG database is a database of numerous metabolic pathways organized in a specific graphical language, which includes pathway information on multiple aspects of genetic processing, cellular processes, metabolism, and organismal systems. Among the KEGG pathway annotation results (Figure 4A), phagosomes and apoptosis had the most DEPs annotated, followed by the pathways of the tight junction, DNA replication, the gap junction, drug metabolism—other enzymes, the regulation of actin cytoskeleton, and the Rap1 signaling pathway. It is hypothesized that Pm-Aps exerts its antimicrobial effects mainly through the pathways of translocation and catabolism, cell growth and death, cell community (eukaryotic), replication and repair, xenobiotic biodegradation and metabolism, cell motility, and signal transduction. The KEGG pathway significance enrichment level of DEPs was further analyzed at *p* < 0.05 and tested with Fisher’s exact test. As shown in Figure 4B, the homologous recombination, DNA replication, and mismatch repair pathways underwent dramatically enriched changes.

### 2.6. Structural Domain Analysis of DEPs

**Figure 5:**
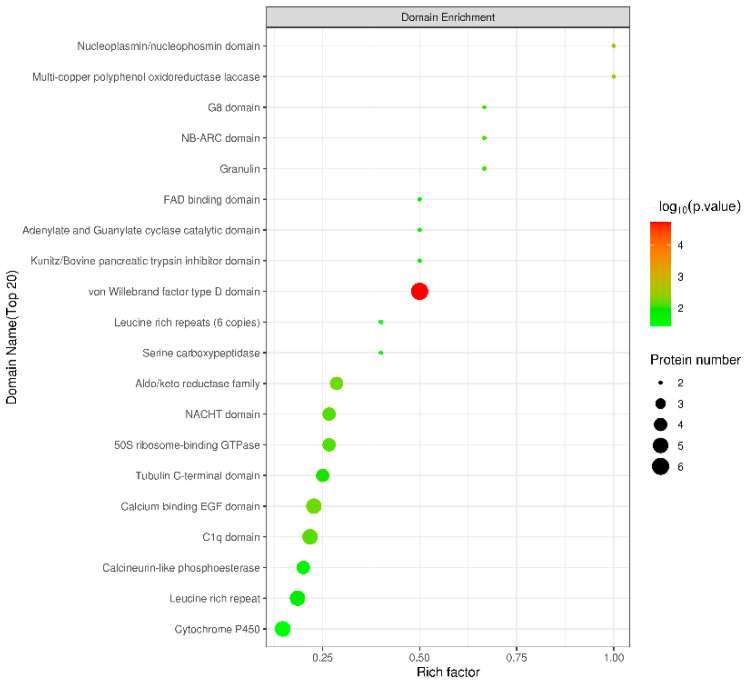
Structural domain enrichment bubble diagram of DEPs. The rich factor is the ratio of the number of relevant differential proteins to the number of all characterized proteins in that structural domain.

Protein structural domains are closely linked by adjacent supersecondary structures in the polypeptide chain, forming two or more localized regions that can be clearly distinguished in space. Different structural domains bear different biological functions. Therefore, structural domain prediction is of great importance for studying the key functional regions of proteins and the potential biological roles they play. In this study, the software Interproscan (Version 5.59-91.0) [25] was used to predict the structural domains of DEPs, and Fisher’s exact test was used for structural domain enrichment analysis. The enrichment results are shown in Figure 5, the structural domain enrichment mainly included the von Willebrand factor type D domain, C1q domain, cytochrome P450, and FAD binding domain.

### 2.7. Effect of Catalase on the Activity of Pm-Aps

H_2_O_2_ is a strong oxidant that can act as a signaling molecule involved in the oxidative quenching of phagocytes, leading to microbial death [26]. The effect of catalase on the activity of Pm-Aps was explored, and the results are shown in Table 4. The circle of inhibition disappeared after the addition of catalase to Pm-Aps, whereas the circle of inhibition was still produced by Pm-Aps after the addition of an equal amount of sterile PBS. It was hypothesized that some effective components in Pm-Aps might inhibit the growth of microorganisms through the production of H_2_O_2_, and the addition of catalase caused the decomposition of H_2_O_2_, which resulted in the disappearance of the inhibitory effect.

### 2.8. Effect of Different Amino Acid Substrates on H_2_O_2_ Production by Pm-Aps

**Figure 6:**
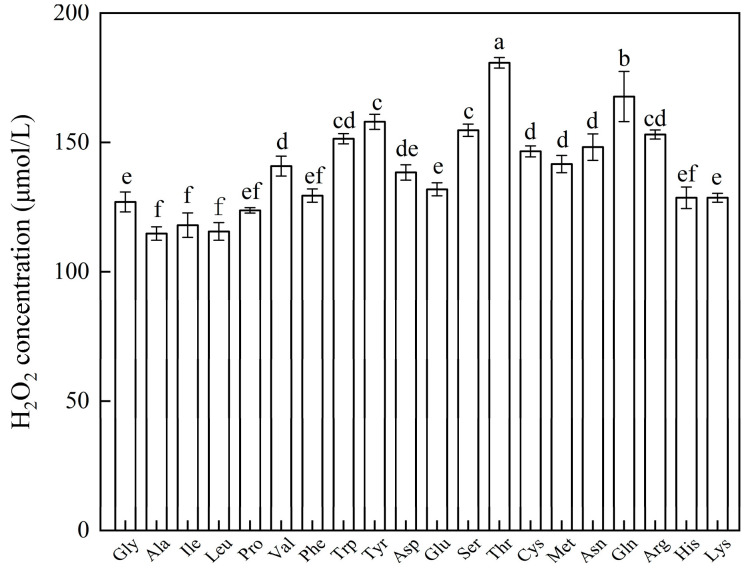
The effect of different amino acid substrates on H_2_O_2_ concentration. Different alphabets indicate significant differences (*p* < 0.05).

The ability of Pm-Aps to generate H_2_O_2_ was further explored using 20 L-type amino acids as substrates. As can be seen in Figure 6, Pm-Aps produced H_2_O_2_ for all amino acid substrates, while no H_2_O_2_ was detected after the addition of catalase (Table S1). It was inferred that Pm-Aps might produce H_2_O_2_ by catalyzing the oxidation of amino acids.

## 3. Discussion

In recent years, the application of natural antimicrobial agents in food processing and aquaculture has received increasing attention. Compared with traditional chemical antimicrobials, novel antimicrobials of natural origin exhibit the advantages of safety, low toxicity, and high activity against drug-resistant bacteria. Therefore, in this study, we utilized VP to stimulate the secretion of Pm-Aps by *Pinctada martensii* to evaluate its antimicrobial effects, identify the main active components, and explore its potential antimicrobial mechanism.

Firstly, we found that Pm-Aps after VP stimulation possessed higher antimicrobial activity, which is the same as the study by Lin [22]. Further exploration of the effect of Pm-Aps revealed broad-spectrum antimicrobial activity, including strong inhibitory activity against VP, LM, SA, BC, EC, PA, SE, and SI. Pm-Aps had the best inhibitory effect on VP, which might be related to the fact that Pm-Aps was induced to be produced by it. Similar results were found in the study by Zhu Xiaoqi [6], who used EC and inactivated EC to induce the production of antimicrobial peptides by *Tenebrio Molitor*. And it was found that the concentration of antimicrobial peptides produced by the induced group was significantly higher than that of the uninduced group; at the same time, the antimicrobial peptides produced by the induction inhibited EC better than SA and *Salmonella*.

Currently, proteomics has been widely used to study the effects of proteins on microorganisms, which helps us to identify DEPs and the relevant functional pathways enriched with them, to mine the core targets of proteins acting on microorganisms, and to understand the relevant mechanisms of action [27]. From the results of proteomics analyses, we found that the expression of L-amino-acid oxidase-like isoform X1 (*LAAO*), alcohol dehydrogenase (*CHDH*), toll-like receptor 4 (*TLR2*), autophagy-related protein 16-1-like (*ATG16L1*), Bcl-2 homologous antagonist/killer (*BAK*), calcium-activated chloride channel regulator 1-like (*CLCA4*), and caspase-8 (*CASP8*) were significantly upregulated in Pm-Aps after VP stimulation. It has been reported that *BAK*, *CASP8*, *CHDH*, and *CLCA4* elevated expression hastens the onset of apoptosis [28,29,30,31,32]. In addition, *TLR2* was involved in the recognition of lipopolysaccharide, flagellum, and cell wall components of many bacteria, which is an important protein in the antimicrobial pathway [33]. The overexpression of *ATG16L1* could accelerate autophagy to inhibit bacterial growth [34]. Notably, *LAAO* participated in the oxidative quenching by phagocytes by catalyzing the oxidative deamination of L-type amino acids to produce H_2_O_2_, which killed bacteria [26]. On the other hand, zygotic DNA replication licensing factor mcm3 (*MCM3*), DNA replication licensing factor mcm5-like (*MCM5*), thymidylate kinase-like (*DTYMK*), serine/threonine-protein kinase PLK1 (*PLK1*), F-box only protein 44-like isoform X2 (*FBXO6*), lysophospholipid acyltransferase 5-like (*LPCAT3*), glutathione S-transferase theta-1 (*GST*), regulator complex protein LAMTOR5 homolog (*LAMTOR5*), steroid 17-alpha-hydroxylase/17,20 lyase-like (*CYP17A*), lysosomal protective protein isoform X1 (*CTSA*), and ribonucleoside-diphosphate reductase large subunit-like (*RRM1*) expression were dramatically downregulated in Pm-Aps. Among them, reduced levels of *PLK1*, *FBXO6*, *LPCAT3*, *LAMTOR5*, *CYP17A*, *MCM3*, *MCM5*, and *DTYMK* were able to promote apoptosis [35,36,37,38,39,40,41]. Previous studies have found that *GST* decreases the antimicrobial effect of antibiotics [42], suggesting that reduced *GST* expression can improve antimicrobial efficacy. Furthermore, *CTSA* and *RRM1* were found to be associated with autophagy, and a reduction in their expression could facilitate the cellular autophagy process [43,44]. These results suggested that the elevated antimicrobial activity of Pm-Aps may be associated with the upregulated expression of *LAAO*, *CHDH*, *TLR2*, *ATG16L1*, *BAK*, *CLCA4*, and *CASP8*. It was also associated with the downregulated expression of *MCM3*, *MCM5*, *DTYMK*, *PLK1*, *FBXO6*, *LPCAT3*, *GST*, *LAMTOR5*, *CYP17A*, *CTSA*, and *RRM1*.

GO functional analysis revealed that DEPs, through cellular processes, regulate P53-like mediator expression, DNA metabolism, and thus apoptosis and autophagy to resist VP infection. Waveform proteins have been found to resist bacterial infection by participating in many cellular processes, including immune signaling, autophagy, and cell adhesion [45], and P53 could regulate apoptosis and autophagy via modulating various signaling pathways [46], which is similar to our study. Generally, abnormalities in DNA replication and DNA repair (including homologous recombination and mismatch repair) cause apoptosis [47,48]. The PI3K-Akt signaling pathway has a role in modulating inflammation, apoptosis, and oxidative stress [49]. Therefore, the results of KEGG pathway analysis showed that Pm-Aps may accelerate bacterial clearance and autophagy by inhibiting homologous recombination, DNA replication, and mismatch repair pathways, which in turn, promote P53-mediated apoptosis and autophagy. Concurrently, Pm-Aps also instigates inflammatory and oxidative stress responses by regulating phagosomes and the PI3K-Akt signaling pathway [50], which catalyzes the cellular production of excessive ROS to damage proteins, nucleic acids, and lipids and activates apoptotic signaling pathways [51] with the objective of inhibiting bacterial growth.

The von Willebrand factor is a multifunctional protein in plasma that interacts with bacteria, promoting bacterial attachment and exacerbating bacterial infections [52]. The overexpression of CYP 450 has been observed to suppress the innate immune response and exacerbate the severity of bacterial infections [53]. In addition, the C1q structural domain is a prototypical lectin structural domain that serves to recognize pathogens and activate immune signaling pathways [54]. Meanwhile, the FAD binding domain is one of the characteristic structural domains of the *LAAO* family [55]. The structural domain enrichment results indicated that, when the organism was infected by VP, Pm-Aps induced the organism to produce a substantial number of immune factors to participate in the immune defense against pathogens. This was achieved by downregulating the protein expression of the von Willebrand factor structural domain and CYP 450 as well as upregulating the protein expression of the C1q structural domain and the FAD binding domain.

Of all the DEPs associated with antimicrobial activity, the highest expression was upregulated by *LAAO*, a common protease in nature that catalyzes the oxidation of L-type amino acids to produce H_2_O_2_ [26], which acts as an intracellular signaling molecule involved in the oxidative burst of phagocytosis, thereby inhibiting bacterial growth [26]. By exploring the effect of catalase on Pm-Aps activity, we found that the antimicrobial activity of Pm-Aps disappeared. In addition, Pm-Aps catalyzed the oxidation of a variety of amino acids to generate H_2_O_2_, but no H_2_O_2_ was detected after the addition of catalase (Table S1). This may be due to the fact that the H_2_O_2_ generated from the oxidation of amino acids catalyzed by the *LAAO* in Pm-Aps was decomposed by catalase, which reduced its antimicrobial activity. A study by Kasai et al. also showed that catalase inhibits the antibacterial activity of *LAAO* [26]. In summary, we speculated that *LAAO* in Pm-Aps might kill bacteria by catalyzing the H_2_O_2_ produced from the oxidation of amino acid substrates.

## 4. Materials and Methods

### 4.1. Strains and Materials

*Vibrio parahaemolyticus* (VP, GDMCC1.306, gram-negative) was provided by the Guangdong Provincial Strain Collection Center (Guangzhou, China). *Listeria monocytogenes* (LM, ATCC19111^T^, gram-positive), *Bacillus cereus* (BC, CICCG3301^T^, gram-positive), *Staphylococcus aureus* (SA, ATCC29213^T^, gram-positive), *Escherichia coli* (EC, ATCC35218^T^, gram-negative), *Pseudomonas aeruginosa* (PA, ATCC27853^T^, gram-negative), *Streptococcus agalactiae* (SE, ATCC12386^T^, gram-positive), and *Streptococcus iniae* (SI, ATCC29178^T^, gram-positive) were acquired from the Food Safety Microbiology Conservation Laboratory, Guangdong Ocean University (Zhanjiang, China). *Pinctada martensii* was sourced from Zhanjiang, China. Penicillin/streptomycin/gentamicin mixed solution (100× triple antibiotic) and kanamycin solution were purchased from Beijing Solepol Technology Co., Ltd. (Beijing, China). Tryptic soy peptone liquid medium (TSB) and plate counting agar medium were offered by Guangdong Huankai Microbial Technology Co., Ltd. (Guangzhou, China). Trifluoroacetic acid (TFA), hydrogen peroxide (H_2_O_2_), formic acid, catalase, 20 amino acids, and acetonitrile were purchased from Shanghai Ron Reagent Co., Ltd. (Shanghai, China). The sterile PBS buffer, H_2_O_2_ content determination kit, and BCA protein concentration kit were provided by Biyuntian Biotechnology Co., Ltd. (Shanghai, China). Trypsin, dithiothreitol (DTT), urea, tetraethylammonium bromide, and iodoacetamide were obtained from Sigma-Aldrich Co., Ltd. (St. Louis, MO, USA).

### 4.2. Strain Activation and Suspension Preparation

VP lyophilized powder was picked and inoculated into TSB (5 mL), which was incubated at 36 °C until turbid, and then the bacterial solution (0.1 mL) was aspirated into sterilized TSB (10 mL) for activation. After the activation was completed, the bacterial solution (2%) was added to the TSB for expansion culture. The resulting bacterial solution was centrifuged to take the precipitate (8000 r/min for 5 min), which was resuspended and diluted to 1 × 10^12^ cfu/mL with sterile PBS to obtain the VP solution [56].

### 4.3. VP Stimulates Infection

We used the method of Lin with minor modifications [22]. *Pinctada martensii* (about 9.5–15.5 cm in shell length, 2.5 years old, from Liusha Bay of Zhanjing City) were rinsed, and the parasitic organisms were removed from their shells for temporary rearing in the preservation room. On the 3rd day of transient rearing, a mixture of antibiotics (0.01%, penicillin/streptomycin/gentamicin/kanamycin mixed solution) was added to the seawater. After 24 h, *Pinctada martensii* were randomly divided into a control group (giC) and an experimental group (giVp). The giVP group was stimulated with closed-shell muscle injections using VP solution (0.2 mL). The giC group was injected using an equal volume of PBS buffer solution. After injection, they were individually transferred to sterilized, clean seawater for a temporary period of 6 h, and the gill tissues were obtained.

### 4.4. Antimicrobial Protein Extraction from Gill Tissue

The gill tissue was homogenized with TFA (0.01%) at a ratio of 1:3 (*w/v*) for 3–5 min; then, the supernatant was centrifuged (8000 r/min, 20min) and lyophilized to obtain the Pm-Aps.

### 4.5. Effect of VP-Stimulated Infection on the Antimicrobial Activity of Pm-Aps

The antimicrobial activity of Pm-Aps was determined using the micro radial diffusion method. A total of 100 μL of 1 × 10^8^ CFU/mL bacteria suspension was pipetted into 10 mL of sterile trypticase soy agar (plus 3% NaCl for Vibrio strains). The mixture was vortexed and then poured into a petri dish. A total of 10 μL of Pm-Aps sample (with a protein concentration of 100 mg/mL) was pipetted into a well with a diameter of about 3.0 mm; sterile PBS was used as a normal control (NC), and 1.5% H_2_O_2_ was used as a positive control (PC). All the test samples were allowed to diffuse at 4 °C for 1 h, and the plates were incubated at 37 °C for 20 h. The diameter of the clear inhibition zone was measured to the nearest 0.1 mm [57].

### 4.6. Determination of Antimicrobial Spectrum and Antimicrobial Activity of Pm-Aps

The antimicrobial spectrum of Pm-Aps was determined by the trace radial diffusion method as in Section 4.5. Referring to the method of Lin [22] with slight modifications, we determined the antimicrobial activity of Pm-Aps using an inhibitory titer (IT, which was defined as the reciprocal of the highest dilution at which bacterial growth is inhibited (24 h of incubation); the greater the IT, the better its antibacterial activity). The bacterial suspension was prepared as in Section 4.2. The Pm-Aps solution (256 mg/mL) was diluted to 128, 64, 32, 16, and 8 mg/mL. A total of 0.18 mL of Pm-Aps solution and TSB medium were added to the sterile 96-well plate, and 0.02 mL of bacterial suspension was added. Among them, the wells without bacterial solution were negative controls. The absorbance was measured at 600 nm after 24 h of incubation at 37 °C in a thermostat. The difference between the absorbance value of the sample group and the absorbance value of the negative control was less than 0.05, which was considered to be an antibacterial effect.

### 4.7. Proteomic Analysis

#### 4.7.1. Sample Pre-Treatment

Pm-Aps were trypsinized using the FASP method, and the peptides were desalted and lyophilized using a C18 cartridge (Empore™ SPE Cartridges C18 (standard density), bed I.D. 7 mm, volume 3 mL, Sigma, St. Louis, MO, USA). The lyophilized samples were reconstituted using a 0.1% formic acid solution, and the peptide content was determined at 562 nm [58].

#### 4.7.2. Liquid Chromatography–Mass Spectrometry/Mass Spectrometry (LC–MS/MS)

LC–MS/MS analysis was performed on a timsTOF Pro mass spectrometer coupled to a Nanoelute (Bruker, Billerica, MA, USA) with a coupling time of 120 min. Samples were uploaded in buffer A (0.1% formic acid) onto a reversed-phase trap column (Thermo Scientific Acclaim PepMap100, 100 μm × 2 cm, nanoViper C18, Waltham, MA, USA), which was coupled to a C18 reversed-phase analytical column (Thermo Scientific Easy Column, 10 cm long, 75 μm ID, 3 μm resin). Separation was performed in a linear gradient with buffer B (84% acetonitrile and 0.1% formic acid) at a flow rate of 300 nL/min. The mass spectrometer was operated in the positive ion mode, and ion mobility mass spectra were collected in the mass range of *m*/*z* 100–1700, 1/k0 of 0.6–1.6, followed by 10 cycles of PASEF MS/MS with a target intensity of 1.5k, a threshold of 2500, and a release time of 0.4 min.

#### 4.7.3. Protein Identification and Quantification

The identification, retrieval, and quantitative analysis of mass spectrometry data were performed using MaxQuant 1.6.14 software. The parameters were as follows: (i) intact tryptic peptide with up to 2 missing cleavage sites; (ii) Carbamidomethyl (C) as a fixed modification; and (iii) Oxidation (M) as a variable modification. The precursor mass tolerance was set to 20 ppm for the initial search and 6 ppm for the primary search, the MS/MS Tolerance was set to 20 ppm, and the false discovery rate (FDR) was 1%. Quantitative analyses were performed using label-free quantification (LFQ) [59].

#### 4.7.4. Bioinformatics Analysis

Protein clustering analysis was performed using the Complexheatmap R package (R Version 3.4) to classify both samples and protein expression dimensions. Protein structural domains were analyzed using the Pfam database (http://pfam.xfam.org/, accessed on 9 September 2023). Blast2GO (http://www.geneontology.org, accessed on 10 September 2023) was used for the Gene Ontology (GO) functional annotation of the target protein collection. The Kyoto Encyclopedia of Genes and Genomes (KEGG) pathway annotation of the target protein collection was performed using KEGG Automatic Annotation Server software (https://www.genome.jp/tools/kaas/, accessed on 10 September 2023).

### 4.8. Determination of the Effect of Catalase on the Antimicrobial Activity of Pm-Aps

The catalase (0.01 g) was dissolved in PBS (1 mL) and mixed well. The antimicrobial activity was determined by the micro radial diffusion method with reference to Section 4.5. Pm-Aps (0.01 mL, 64 mg/mL) and PBS (4 μL) were added to the sample group, while Pm-Aps (0.01 mL, 64 mg/mL) and catalase (4 μL, 10 mg/mL) were added to the control group [60].

### 4.9. The Effect of Different Amino Acid Substrates on H_2_O_2_ Production by Pm-Aps

Pm-Aps solution (25 μL, 64 mg/mL), amino acid solution (25 μL, 20 mmol/L), and PBS (25 μL) were mixed as the sample group, and the control group was added with Pm-Aps solution (25 μL, 64 mg/mL), amino acid solution (25 μL, 20 mmol/L), and catalase (25 μL, 10 mg/mL), while the H_2_O_2_ concentration resulting from the reaction was measured according to the H_2_O_2_ content determination kit instructions [61].

### 4.10. Statistical Analysis

All data were replicated three times, and results were expressed as mean ± standard deviation (SD). Graphs were plotted using Graphpad Prism 9.0, and experimental data were analyzed by one-way ANOVA with post hoc comparisons using Duncan’s method using SPSS 26.0 software. *p* < 0.05 was considered significant.

## 5. Conclusions

In this study, we used VP to stimulate *Pinctada martensii* and extracted Pm-Aps from gill tissues to determine their antimicrobial activities. It was found that the antimicrobial activity of Pm-Aps was significantly elevated after VP stimulation, and it had high broad-spectrum antimicrobial activity. Proteomic analysis showed that enhanced Pm-Aps activity was mainly associated with the upregulation of the expression of *LAAO*, *CHDH*, *TLR2*, *ATG16L1*, *BAK*, *CLCA4*, and *CASP8* as well as the downregulation of the expression of *MCM3*, *MCM5*, *DTYMK*, *PLK1*, *FBXO6*, *LPCAT3*, *GST*, *LAMTOR5*, *CYP17A*, *CTSA*, and *RRM1*. It is hypothesized that the related proteins inhibit bacterial growth and reproduction by hindering DNA replication and repair, inducing apoptosis and autophagy, and activating immune-related signaling pathways. In addition, we found that the key component of Pm-Aps exerting antimicrobial effects is *LAAO*, which exerts bactericidal effects by catalyzing the oxidation of amino acids to generate H_2_O_2_. In conclusion, these findings provide some theoretical basis for the development of Pm-Aps.

However, the current study still has some limitations. The proteomics results still need to be further validated and analyzed, and Pm-Aps need to be isolated and purified. Thus, in the next step, the proteomics results (DEPs) can be validated using methods such as the Quantitative Polymerase Chain Reaction (qPCR) and the Western Blot (WB). Secondly, *LAAO* was obtained by isolation and purification of Pm-Aps using sodium dodecyl sulfate-polyacrylamide gel electrophoresis (SDS-PAGE). In addition, we can also obtain the full length of the gene of *LAAO* in Pm-Aps by cloning, and the full length of its cDNA sequence could be analyzed by bioinformatics, which is expected to prepare recombinant *LAAO* efficiently by constructing prokaryotic and eukaryotic expression systems.

## Figures and Tables

**Table 1:** Antimicrobial spectrum and antimicrobial activity of Pm-Aps.

Strain	Diameter (mm)	IT
*Vibrio parahaemolyticus* (VP)	11.51 ± 0.54	16
*Listeria monocytogenes* (LM)	7.40 ± 0.10	8
*Staphylococcus aureus* (SA)	7.50 ± 0.10	8
*Bacillus cereus* (BC)	5.43 ± 0.05	4
*Escherichia coli* (EC)	4.23 ± 0.05	4
*Pseudomonas aeruginosa* (PA)	5.30 ± 0.10	4
*Streptococcus agalactiae* (SE)	6.37 ± 0.11	4
*Streptococcus iniae* (SI)	5.20 ± 0.10	4

Inhibitory titer (IT) was defined as the reciprocal of the highest dilution to inhibit the growth of bacteria (incubated for 24 h); the greater the IT, the higher the inhibitory activity.

**Table 2:** Key DEPs with upregulated expression.

Protein	Gene	Description	giVp/giC (FC)
TR14358_c0_g1_ORF	*LAAO*	L-amino-acid oxidase-like isoform X1	4.670
TR24434_c0_g1_ORF_1	*CHDH*	alcohol dehydrogenase	4.278
TR7325_c1_g1_ORF	*TLR2*	toll-like receptor 4	4.234
TR38222_c0_g1_ORF	*ATG16L1*	autophagy-related protein 16-1-like	3.554
TR7164_c0_g1_ORF	*BAK*	Bcl-2 homologous antagonist/killer	2.460
TR1646_c0_g1_ORF	*CLCA4*	calcium-activated chloride channel regulator 1-like	2.282
TR775_c1_g1_ORF	*CASP8*	caspase-8	2.086

**Table 3:** Key DEPs with downregulated expression.

Protein	Gene	Description	giVp/giC (FC)
TR10572_c0_g1_ORF	*MCM3*	zygotic DNA replication licensing factor mcm3	0.469
TR4065_c0_g1_ORF	*MCM5*	DNA replication licensing factor mcm5-like	0.466
TR73065_c0_g2_ORF	*DTYMK*	thymidylate kinase-like	0.369
TR3406_c0_g1_ORF	*PLK1*	serine/threonine-protein kinase PLK1	0.351
TR20414_c0_g1_ORF	*FBXO6*	F-box only protein 44-like isoform X2	0.329
TR6309_c1_g2_ORF	*LPCAT3*	lysophospholipid acyltransferase 5-like	0.308
TR13088_c0_g1_ORF	*GST*	glutathione S-transferase theta-1	0.281
TR41412_c0_g2_ORF	*LAMTOR5*	regulator complex protein LAMTOR5 homolog	0.263
TR8221_c0_g1_ORF	*CYP17A*	steroid 17-alpha-hydroxylase/17,20 lyase-like	0.152
TR19429_c0_g1_ORF	*CTSA*	lysosomal protective protein isoform X1	0.090
TR16486_c0_g1_ORF	*RRM1*	ribonucleoside-diphosphate reductase large subunit-like	0.015

**Table 4:** The effect of catalase on the antimicrobial activity of Pm-Aps.

Strain	Diameter (mm)	
	Pm-Aps and PBS	Pm-Aps and Catalase
*Vibrio parahaemolyticus* (VP)	10.13 ± 0.05	-
*Listeria monocytogenes* (LM)	6.20 ± 0.10	-
*Staphylococcus aureus* (SA)	5.83 ± 0.05	-
*Bacillus cereus* (BC)	4.50 ± 0.10	-
*Escherichia coli* (EC)	3.53 ± 0.05	-
*Pseudomonas aeruginosa* (PA)	4.20 ± 0.10	-
*Streptococcus agalactiae* (SE)	5.33 ± 0.15	-
*Streptococcus iniae* (SI)	4.26 ± 0.05	-

“-” indicates no antimicrobial activity.

## Data Availability

Data are available upon request.

## Supplementary Materials

The following supporting information can be downloaded at: https://www.mdpi.com/article/10.3390/antibiotics13111100/s1. Table S1: Levels of H_2_O_2_ production by different amino acid substrates.
