# Supplementary material for: Proteomic Analysis of Vibrio parahaemolyticus-Stimulated Pinctada martensii Proteins for Antimicrobial Activity, Potential Mechanisms, and Key Components
Source: Antibiotics (Basel). 2024 Nov 19;13(11):1100. doi: 10.3390/antibiotics13111100 (PMC11590882; doi:10.3390/antibiotics13111100)
Supplement: Supplementary file 1 [file antibiotics-13-01100-s001.zip › antibiotics-3239648-supplementary.pdf]

Table S1. Levels of H_2O_2 production by different amino acid substrates.

**Amino acid**	**H_2O_2 concentration (μmol/L)**	
	Pm-Aps and PBS	Pm-Aps and catalase
Gly	126.98±3.86^{e}	-
Ala	114.75±2.60^{f}	-
Ile	118.01±4.70^{f}	-
Leu	115.57±3.42^{f}	-
Pro	123.72±1.06^{ef}	-
Val	140.83±3.84^{d}	-
Phe	129.42±2.58^{ef}	-
Trp	151.43±1.99^{cd}	-
Tyr	157.95±2.92^{c}	-
Asp	138.39±2.98^{de}	-
Glu	131.87±2.52^{e}	-
Ser	154.69±2.40^{c}	-
Thr	180.77±2.02^{a}	-
Cys	146.54±2.13^{d}	-
Met	141.65±3.35^{d}	-
Asn	148.17±5.14^{d}	-
Gln	167.73±9.68^{b}	-
Arg	153.06±1.74^{cd}	-
His	128.61±4.15^{ef}	-
Lys	128.61±1.72^{e}	-

"-" indicates the absence of H_2O_2. Different alphabets indicate significant differences ($p < 0.05$).
